# Spatial distribution of *Ixodes ricinus* in forest habitats: a comparative study of the northern and southern slopes of Mount Slavnik, Slovenia

**DOI:** 10.1051/parasite/2025044

**Published:** 2025-07-25

**Authors:** Jure Jugovic, Alenka Babič, Anka Kuhelj, Vladimir Ivović

**Affiliations:** Department of Biodiversity, Faculty of Mathematics, Natural Sciences and Information Technologies, University of Primorska Glagoljaška 8 6000 Koper Slovenia

**Keywords:** Ticks, Elevational distribution, Slope orientation, Temperature, Relative humidity

## Abstract

While previous studies have examined the elevational limits of *Ixodes ricinus* in Central Europe, this study is the first to investigate the influence of slope orientation on tick distribution in Slovenia. Our results provide new insights into how two important microclimatic factors, temperature and humidity, vary between the northern and southern slopes, and how these differences affect the abundance of *I. ricinus*, a factor that has not been studied in this region before. We found that nymph density was highest on the southern slope at intermediate elevations (720 m) and increased with temperature. In contrast, fewer adult ticks were found on the southern slope compared to the northern slope, most of them at 720 m elevations. The significantly higher abundance of adult ticks on the northern slope is probably related to the cooler temperatures, higher relative humidity, denser ground vegetation and greater availability of hosts such as roe deer. Although these results are regionally specific, they contribute to a more comprehensive understanding of the ecological factors influencing the distribution of *I. ricinus* in Central Europe.

## Abstract (fr)

Des études antérieures ont examiné les limites d’altitude d’*Ixodes ricinus* en Europe centrale, mais cette étude est la première à examiner l’influence de l’orientation des pentes sur la répartition des tiques en Slovénie. Nos résultats apportent de nouvelles informations sur la façon dont deux facteurs microclimatiques importants, la température et l’humidité, varient entre les versants nord et sud, et sur l’impact de ces différences sur l’abondance d’*I. ricinus*, un facteur qui n’avait jamais été étudié dans cette région auparavant. Nous avons constaté que la densité de nymphes était maximale sur le versant sud, à des altitudes intermédiaires (720 m), et qu’elle augmentait avec la température. En revanche, moins de tiques adultes ont été trouvées sur le versant sud que sur le versant nord, la plupart à 720 m d’altitude. L’abondance significativement plus élevée de tiques adultes sur le versant nord est probablement liée aux températures plus fraîches, à une humidité relative plus élevée, à une végétation au sol plus dense et à une plus grande disponibilité d’hôtes tels que les chevreuils. Bien que ces résultats soient spécifiques à chaque région, ils contribuent à une compréhension plus complète des facteurs écologiques influençant la distribution d’*I. ricinus* en Europe centrale.

## Introduction

Ticks (Acari: Ixodida) are obligate haematophagous ectoparasites [5, 22] that transmit a variety of pathogens [15, 23], including *Borrelia burgdorferi* (the causative agent of Lyme disease), tick-borne encephalitis virus and *Babesia* spp. [31, 32]. Environmental factors such as climate change, host population dynamics and urbanisation are altering the geographical distribution and temporal activity patterns of tick species, increasing the incidence and spread of tick-borne diseases.

Ticks belong to three families: hard ticks (Ixodidae), soft ticks (Argasidae), and the non-European Nuttalliellidae [14], whereby the European ticks are classified into seven genera. Of the more than 900 tick species currently described, around 70 tick species are found in Europe [17, 34]. So far, 16 tick species have been documented in Slovenia [43, 44, 45, 46, 47, 48, 49, 50, 51]. The most common tick species in Slovenia as well as in Europe is *I. ricinus*. Two other species, *Dermacentor reticulatus* Fabricius, 1794 and *Haemaphysalis concinna* C. L. Koch, 1844, were only recorded in the north-eastern part of the country between 2005 and 2008 [26]. Depending on the geographical area, *I. ricinus* can show unimodal or bimodal annual population dynamics, while in some areas both are possible depending on the location [37]. In Slovenia, this species shows a bimodal seasonal activity pattern with a peak in spring (April, May, June) and another in autumn (September or October) [26]. Older studies on ticks in Slovenia, conducted between 1954 and 1979, showed that small mammal hosts such as the European polecat (*Mustela putorius*) and the northern white-breasted hedgehog (*Erinaceus roumanicus*) were frequently infested by two species of the genus *Ixodes*: *I. ricinus* and *I. hexagonus* Leach, 1815 [44].

Many factors play a role in the elevational spread of the important vector tick *I. ricinus* in Europe [30]. These factors can be categorised as climatic (temperature, precipitation, snow cover), ecological (vegetation period, habitat structure, presence of hosts), anthropogenic (habitat structure, wildlife management, land use patterns, forestry), and others (orientation of slopes). Although these factors are closely linked, they often cannot be quantified precisely. However, the evidence pointing to a change in the elevational distribution of *I. ricinus* has already been discussed [30]. The modes of action associated with changes in the elevational distribution of *I. ricinus* are: increased overall and winter temperatures [16, 33], snow cover [12, 30], wildlife and forest management, and orientation of mountain slopes [30].

The activity of *I. ricinus* is strongly influenced by temperature and humidity, with the main activity occurring in the warmer months [20, 25, 30]. Desiccation poses a significant threat to all developmental stages of this species, meaning that temperature and humidity play a crucial role in limiting its distribution and activity [41]. In general, *I. ricinus* favours habitats that provide shade and protection from desiccation, such as forests, shrubs and dense grass, although preferences may vary by region [29]. A study conducted in Switzerland on a south-facing mountain found that the density of *I. ricinus*, including nymphs and adults, decreased with elevation. This trend was observed at elevations of 620 m, 740 m and 900 m [24]. However, there are no studies comparing the abundance of ticks on southern and northern slopes.

Ticks are very sensitive to environmental conditions, so that microclimatic factors such as temperature, humidity and vegetation are decisive for their distribution. As south- and north-facing slopes differ in these factors, we assumed that tick density would also vary according to slope orientation. In the Northern Hemisphere, south-facing slopes receive more direct sunlight, resulting in warmer and drier conditions, while north-facing slopes are cooler and more humid due to less sunlight.

This study is the first in Slovenia to examine how slope orientation affects tick distribution. It builds on previous research that investigated the altitudinal limits of *I. ricinus* in Central Europe. Our findings highlight how temperature and humidity differences between north- and south-facing slopes influence tick abundance – a factor that has not been studied in this region before.

## Materials and methods

The planning and execution of the study, along with the processing of all results, were carried out by all authors of the manuscript, both in the field and in the laboratory of the Zoological Studies at the Department of Biodiversity (Faculty of Mathematics, Natural Sciences and Information Technologies, University of Primorska, Izola, Slovenia).

### Study area and sampling sites

Mount Slavnik with its 1028 m high peak is a NW–SE orientated mountain and is located in the northern part of the Dinaric Alps along the Adriatic coast [36]. As it is located in the hinterland of the coastal town of Koper, it is also a popular hiking destination in the Coastal-Karst region of Slovenia.

Slavnik is located in a unique transition zone where the ecological influences of the Mediterranean, Central Europe, the Dinarides, and the Alps meet and produce diverse and complex flora and fauna. The extent of these influences varies with both distance from the coast and elevation [36]. The Slavnik landscape is characterised by grasslands and montane forms of the *Carici humilis – Centaureetum rupestris* plant communities, which are mainly found in (former) pasture areas. These pastures are considered some of the floristically richest areas in Europe, where a mixture of Mediterranean, Mediterranean-montane, Illyrian and Central European plant species thrive [6, 7].

On the cooler, northern slopes that descend towards the villages of Skadanščina and Materija, rich mixed beech forests (*Fagus sylvatica*), especially the *Seslerio autumnalis – Fagetum* communities, are predominant. The sunnier and warmer southern slopes above the village of Podgorje, on the other hand, are characterised by a sub-Mediterranean mixed forest. This forest type is dominated by downy oaks (*Quercus pubescens*) and European hop hornbeams (*Ostrya carpinifolia*), which belong to the *Seslerio autumnalis – Ostryetum* community [6], which emphasises the diverse botanical richness and ecological importance of the region.

Based on unpublished data from the local Slavnik-Materija hunting society and the database of the Slovenian Museum of Natural History (PMS, Ljubljana 2014, https://www.pms-lj.si/en/), large mammals such as roe deer (*Capreolus capreolus*), wild boar (*Sus scrofa*), and foxes (*Vulpes vulpes*) are regularly present on Slavnik, with occasional sightings of brown bears (*Ursus arctos*), golden jackals (*Canis aureus*), and wolves (*Canis lupus*). Smaller mammals include martens (*Martes* spp.), badgers (*Meles meles*), and several species of smaller rodents. Other potential tick hosts in the area include various species of birds, both domestic and migratory, as well as lizards and several species of non-venomous snakes. Additionally, the region has rich entomofauna, further contributing to its biodiversity.

**Figure 1:**
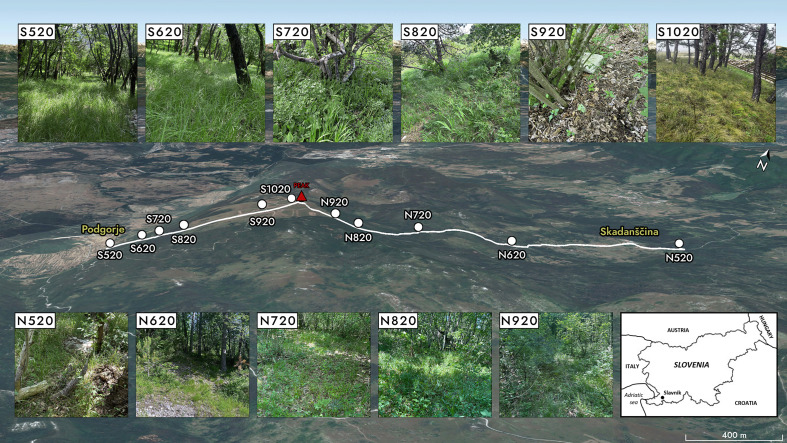
Geographic position of mount Slavnik (inset) with denoted 11 sampling sites of *Ixodes ricinus* along north – (N) and south – (S) facing slopes above villages Skadanščina and Podgorje, respectively, located at each 100 m of elevation (numbers denote elevation in m a.s.l.) in (patches of) a mixed forest. At the highest sampling site, only a small *Pinus nigra* stand on the south-facing slope below the peak (S1020) resembles forest habitat.

A total of eleven collection sites were selected prior to sampling (Table 1 and Fig. 1). Five sampling sites were established at an elevation interval of 100 m each (520 m, 620 m, 720 m, 820 m, 920 m) both from the north-east (near the village of Skadanščina) and from the south-west (near the village of Podgorje). The elevation of each site was precisely determined using a Garmin Montana 750i device (Garmin Ltd., Olathe, KA, USA). The eleventh sampling site was located just below the summit, at an elevation of 1020 m above sea level, in a south-westerly direction. The sampling site at 920 m on the southern slope (S920) was close to the tree line and the highest sampling site (S1020) was in a small, isolated, and sparse *Pinus nigra* stand, both open to a neighbouring meadow exposed to the strong local bora wind (strongest at the top of the windward side of the slope) [35]. Primarily, we chose forest habitats for each site, and moreover, with the exception of the highest 1 020 m site, all other sites were located within the same habitat type, namely mixed forest.

**Table 1:** Summary data on geographic position, habitat, and numbers of collected ticks (Ixodes ricinus), and number of visits (V) at eleven sampling sites (N – north-facing slope; S – south-facing slope; number denotes elevation in metres) on Mount Slavnik (average/median densities per 100 m^2^ added in parentheses). Abundance of leaf litter: scarce = some patches of leaf litter between ground vegetation, thick = litter present all over the site, very thick = mostly thick layer of litter present all over the site. Vegetation height measured at every sampling occasion for each drag, presented by an average value truncated to the nearest 5 cm or range, when it was not homogenous in time. For larvae, exact dates of recordings are shown.

Site	V	Geographic coordinates [°]	Habitat	Prevailing ground vegetation and characteristics	Vegetation height (cm)	Total number of ticks (nymphs + adults)	Larvae (presence)	Nymphs	Males	Females
N520	4	45.549032, 14.0155	Mixed forest	Grass, thick layer of leaf litter, few small rocks	30–40	64 (16.0/19.5)		43 (10.8/13.5)	12 (3.0/3.0)	9 (2.3/3.0)
N620	4	45.540036, 13.9981	Light mixed forest	Needle leaves, thick layer of leaf litter, grass, small rocks	20–30	43 (10.8/10.5)		35 (8.8/9.5)	5 (1.3/1.0)	3 (0.8/0.5)
N720	4	45.538759, 13.9870	Dense mixed forest	Grass, shrubs, thick layer of leaf litter and fallen trees	≈ 30	136 (34.0/40.5)		88 (22.0/22.5)	23 (5.8/6.0)	25 (6.3/7.5)
N820	4	45.536188, 13.9829	Dense mixed forest	Grass, shrubs, thick layer of leaf litter and fallen trees	≈ 30	42 (10.5/8.0)		35 (8.8/7.0)	4 (1.0/1.0)	3 (0.8/0.5)
N920	4	45.532344, 13.9808	Dense mixed forest	Grass, shrubs, thick layer of leaf litter	30–40	32 (8.0/8.5)		24 (6.0/6.0)	6 (1.5/1.5)	2 (0.5/0.0)
S1020	5	45.533747, 13.9756	*Pinus nigra* stand	Grass, some rocks, scarce leaf litter	15–20	1 (0.2/0.0)		1 (0.2/0.0)	0 (0.0/0.0)	0 (0.0/0.0)
S920	5	45.52953, 13.97071	Dense mixed forest and shrubs	Low shrubs, very thick layer of leaf litter, wood litter, few rocks, almost no ground vegetation	≈ 35	194 (38.8/1.0)	5 July	188 (37.6/1.0)	1 (0.2/0.0)	5 (1.0/0.0)
S820	5	45.52741, 13.96622	Light mixed forest	Grass, thick layer of leaf litter	10–20	108 (21.6/12.0)	27 April	98 (19.6/6.0)	5 (1.0/1.0)	5 (1.0/0.0)
S720	5	45.52805, 13.96165	Light mixed forest	Grass, scarce leaf litter	45–50	81 (16.2/8.0)	27 April	54 (10.8/6.0)	14 (2.8/2.0)	13 (2.6/2.0)
S620	5	45.53004, 13.95742	Light mixed forest	Grass, some rocks, scarce Leaf litter	≈ 50	28 (5.6/6.0)		21 (4.2/4.0)	1 (0.2/0.0)	6 (1.2/1.0)
S520	5	45.52963, 13.95305	Light mixed forest	Grass, scarce leaf litter and rocks	40–50	20 (4.0/1.0)		18 (3.6/1.0)	1 (0.2/0.0)	1 (0.2/0.0)
					SUM	749 (15.0/6.5)		605 (12.1/4.5)	72 (1.4/1.0)	72 (1.4/0.0)

### Tick sampling

Previous studies have shown that the peak of seasonal tick activity in the Slovenian Karst coastal region is in spring and early summer [42]. For this reason, tick samples were collected four times on the northern and five times on the southern slope from 27 April to 5 July 2023 (27 April, 3 June, 15 June, 28 June and 5 July) at specific elevations (Table 1). Sampling took place in clear weather and air temperatures above 15 °C. For each sampling event (*i.e.* a day of collecting ticks), two pairs of researchers started sampling at 9 am local time at the two lowest sites (N520 and S520) and continued sampling at successive sampling sites towards the summit. We were always finished by 2 pm local time. The sole exception to this was the first sampling event on 27 April, when northern sites (N520–N920) were not sampled.

A 1 m × 1 m white flannel cloth was used to collect ticks by dragging it on both sides of the hiking trails. In areas with dense vegetation, the cloth was dragged across nearby grassy areas instead. The cloth was dragged over the vegetation and covered a total area of 100 square metres. This was achieved by 20 drags of 5 m each. After each 5 m drag, the cloth was inspected and any adult ticks or nymphs crawling on it were collected. The ticks were then carefully removed from the flag and stored alive in sterile tubes. In the laboratory, the species, stage, and sex of the ticks were determined using the taxonomic key “Ticks of Europe and North Africa” [17].

### Meteorological data

Environmental data were systematically collected throughout the study period using data loggers (HOBO Onset 1-800, Bourne, MA, USA) placed approximately 10 cm above the ground in the vegetation at each sampling site. These loggers were programmed to record relative humidity (RH: %) and temperature (*T*: °C) data once per hour.

### Data analysis

The relative humidity and temperature data from the data loggers were tested for possible significant differences between the both slopes (One-Way T-test) and sampling sites (One-Way ANOVA test), and pairwise comparisons with Bonferroni correction were also performed. Significant differences were accepted at *p* < 0.05.

Only ticks belonging to *I. ricinus* were included in the data analysis. Ticks were counted and divided by sampling site, sex, and stage (nymphs, adult males, and adult females). Larvae were not collected, but their presence was recorded.

We tested for possible differences in the mean (median) number of ticks collected (separately for nymphs and adults) between the north and south facing slope (Mann–Whitney U test). Significant differences were accepted at *p* < 0.05. For each sampling site, also average value of collected ticks (males, females, adults, nymphs, total) was calculated.

Prior to each sampling, temperature and relative humidity were recorded for 24 h (corresponding to 24 records). Average values were used in further analysis.

To test whether side, elevation, temperature, and relative humidity have an impact on tick count, we fitted generalised additive models (GAM) with a negative binomial family from the “mgcv” package [52]. We fitted full models for number of nymphs, adults, and additionally males and females. We also tested elevation, temperature and relative humidity for interaction with side of the slope, temperature and relative humidity with elevation and relative humidity with temperature. We chose the best model by stepwise backward elimination strategy and the Akaike Information Criterion (AIC) criterion. Plots were created with “visreg” package [3].

We conducted all statistical modelling and plotting in R version 4.3.X [39] and run in RStudio [38] interface. For other statistical analyses, SPSS statistical package ver. 20.0 for Windows (IBM SPSS Inc., Armonk, NY, 1989, 2011) was used.

## Results

**Figure 2:**
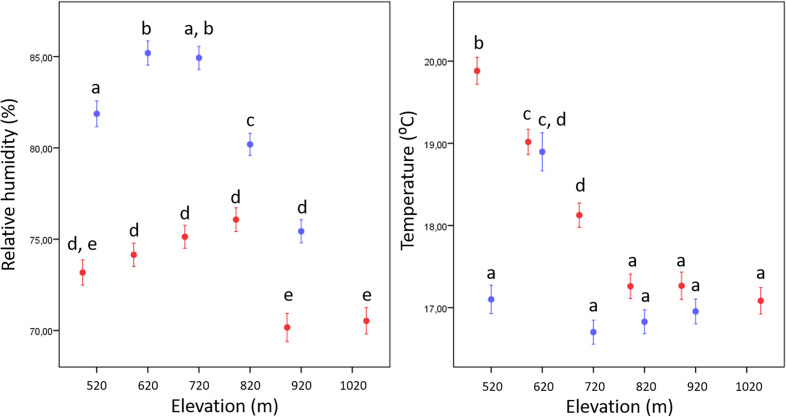
Average relative humidity and temperature (error bars: ± 1 standard error) at eleven sampling sites of *Ixodes ricinus* on north-facing (blue) and south-facing (red) slopes of mount Slavnik in spring-summer 2023. Different letters denote significantly different average values between the sites (one-way ANOVA, *post hoc* pairwise comparisons with Bonferroni correction: *p* < 0.05).

The average relative humidity was higher on the northern slope, with values around 82% (AVG ± SE = 81.5% ± 0.30%, SD = 18.53%, *n* = 3 920), compared to approximately 73% (73.2% ± 0.28%, SD = 19.36%, *n* = 4 704) on the southern slope (N > S). At the three lowest sampling sites (N520–N720 m) on the northern slope and four on the southern slope (S520–S820 m), the average relative humidity was highest and significantly higher than at the higher sampling sites on the same side (Fig. 2, Supplementary File A). The average relative humidity was lowest at the highest sampling site S1020 (AVG ± SE: 70.5% ± 0.72%, SD = 20.22%, *n* = 784). In each pair of two sampling sites at the same elevation, humidity was always higher on the northern slope (Supplementary File A).

The average temperatures measured on the north- and south-facing slopes (N < S) were 17.3 °C (AVG ± SE = 17.30 °C ± 0.08 °C, SD = 4.90 °C, *n* = 3 920) and 18.2 °C (with S1020 = 18.11 °C ± 0.01 °C, SD = 4.52 °C, *n* = 4 704; without S1020: 18.31 °C ± 0.01 °C, SD = 4.49 °C, *n* = 3 920), respectively. The average temperature decreased gradually from the lowest (S520: 19.88 °C ± 0.16 °C, SD = 4.62 °C, *n* = 784) to the highest sampling point (S1020: 17.08 °C ± 0.16 °C, SD = 4.52 °C, *n* = 784) on the southern slope, while it is highest on the northern slope at 620 m elevation (N620: 18.90 °C ± 0.16 °C, SD = 6.48 °C, *n* = 784) with a hiking trail there being wide and open (see Fig. 1). Apart from this elevation (where both sides differ only insignificantly; S620: 19.02 °C ± 0.15 °C, SD = 4.32 °C, *n* = 784), the average temperature on the north side of the slope was always lower than on the south side; however, this difference becomes insignificant at elevation of 820 m and above it (Supplementary File A). While on the southern slope of Slavnik, the average relative humidity was always below 80% during the study period (*i.e.* about 70–75%), but increased with increasing elevation up to 820 m; the decrease in relative humidity was considerable at the two highest sites on this slope (920 m and 1020 m; Fig. 2, Supplementary File A).

A total of 749 unfed specimens of *I. ricinus* were collected, including 605 nymphs, 72 males and 72 females (Table 1). In addition, 5 nymphs of the *Rhipicephalus sanguineus* complex [4] were found, a species previously documented in Slovenia only in coastal regions [15]. Adults were collected at all sampling sites, except for the sampling site at 1 020 meters elevation (closest to the mountain peak), where in total only one nymph was collected.

**Figure 3:**
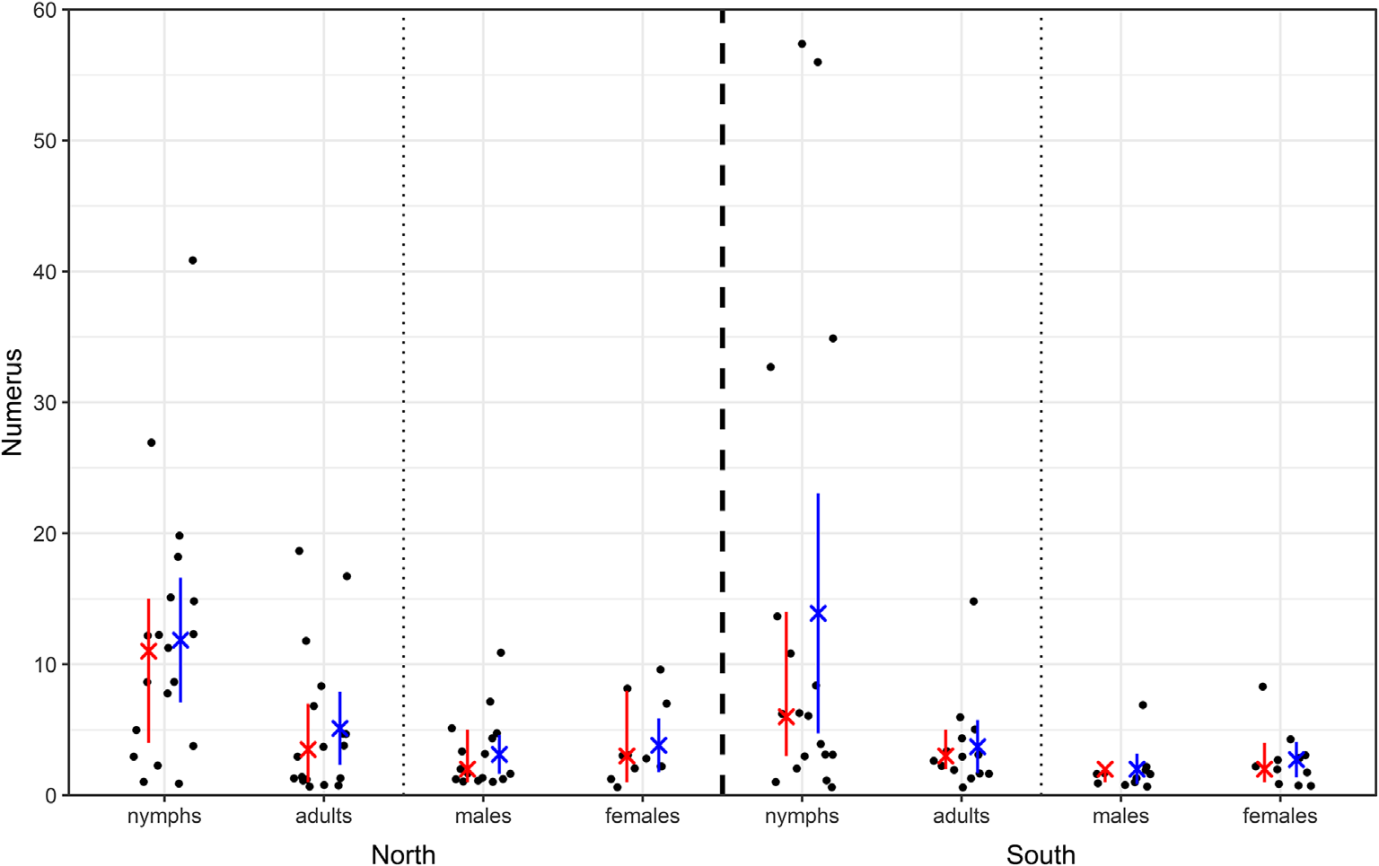
Collected *I. ricinus* ticks on the north and south side. Scale is cropped at 60 (one data point is not shown – 130 for nymphs on the south side). The median and its 95% confidence interval are shown in red, while the mean and its 95% confidence interval are shown in blue.

Mean value (average/median) of collected ticks (nymphs and adults combined) per sampling occasion × site was 15.0/6.5 individuals (*i.e.* per 100 m^2^ of sampling area, total data set; SE = 3.3; SD = 23.5; min–max = 0–133; 25–75 percentiles = 1.0–17.5). The respective value for adults was 2.88/1.0 ticks (SE = 0.62; min–max = 0–19; 25–75 percentiles = 0.0–4.0). In total, median value of collected ticks (Mann–Whitney U test; adults: U = 169.0, Z = −2.670, *p* = 0.008, and nymphs: U = 185.0, Z = −2.296, *p* = 0.022) were lower at the southern than northern side of the slope (Fig. 3).

**Figure 4:**
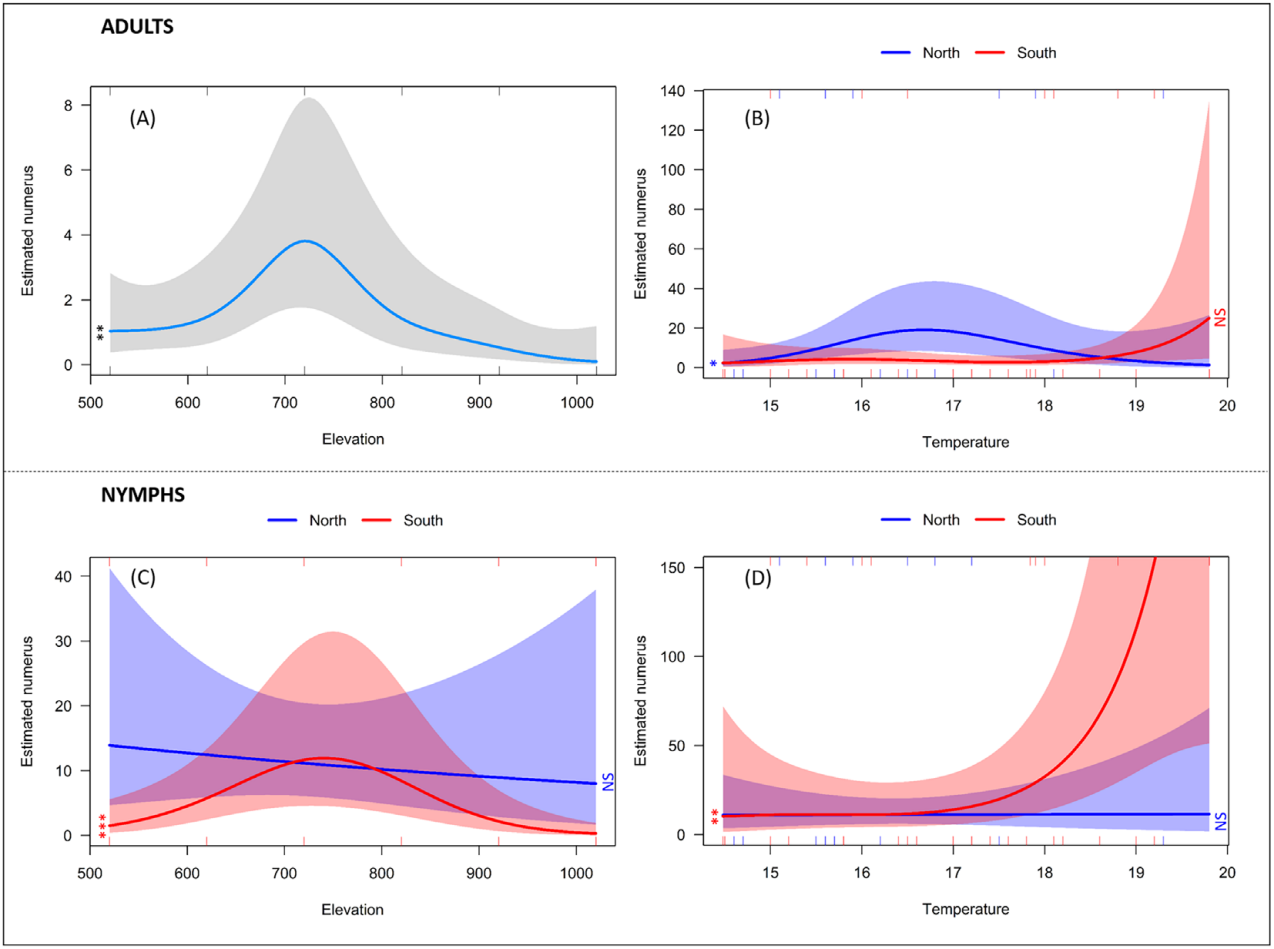
Smooth terms of a negative binomial generalised additive model for abundance of *I. ricinus* adults (A, B) and nymphs (C, D): (A) elevation, (C) elevation on the south and north slopes, (B, D) temperature on the south and north slopes (in D, scale y cropped at 150 for temperature). ***p < 0.001, **p < 0.01, *p < 0.05, NS non-significant.

Chosen generalised additive model (GAM) with a negative binomial family for adult tick abundance explains a substantial portion of the variability in adult tick abundance (adjusted *R*^2^ = 0.61, deviance explained = 59.2%; Table 2, Fig. 4). The model suggests that adult tick abundance is lower on south-facing slopes compared to north-facing slopes (coefficient = −0.774, *p* = 0.026). Elevation has a significant, non-linear effect on tick abundance, with highest abundance of adults at 720 m (coefficient = 3.648, *p* = 0.002, Fig. 4). Temperature has a significant, non-linear effect on tick abundance specifically for north-facing slopes (highest abundance at temperatures 16–17 °C; coefficient = 2.332, *p* = 0.034, Fig. 4), while it does not significantly affect tick abundance on south-facing slopes in a non-linear manner (coefficient = 2.452, *p* = 0.128).

**Table 2:** Chosen generalised additive models (GAM) with a negative binomial family for adults and nymphs showing the interaction effect between side of the slope, elevation, temperature and relative humidity in terms of number of collected adult *Ixodes ricinus*. Significant *p* values (*p* < 0.05) in bold. *** *p* < 0.001, ** *p* < 0.01, * *p* < 0.05, NS non-significant.

	Parametric coefficients	Estimate	Std. error	*z*-Value	*p*	
Adults						
	Intercept	0.822	0.278	2.960	**0.003**	**
	Side of slope – South	−0.774	0.3477	−2.226	**0.026**	*
	*Approx. significance of smooth terms*	*edf*	*Ref. df*	*χ* ^*2*^	*p*	
	Elevation	3.648	4.308	16.930	**0.002**	**
	Temperature × Side of slope − North	2.332	2.705	7.805	**0.034**	*
	Temperature × Side of slope − South	2.452	2.796	6.273	0.128	NS
Nymphs						
	Intercept	2.381	0.332	7.177	**<0.001**	***
	Side of slope – South	−0.628	0.436	−1.442	0.149	NS
	*Approx. significance of smooth terms*	*edf*	*Ref. df*	*χ* ^*2*^	*p*	
	Elevation × Side of slope − North	1.000	1.000	0.213	0.644	NS
	Elevation × Side of slope − South	1.934	1.994	13.658	**<0.001**	***
	Temperature × Side of slope − North	1.000	1.001	0.002	0.971	NS
	Temperature × Side of slope − South	2.167	2.714	11.558	**0.007**	**

Adults: *R*^2^ (adj) = 0.61, Deviance explained = 59.2%, REML = 97.938, Scale est. = 1, *n =* 50; Nymphs: *R*^2^ (adj) = − 0.351, Deviance explained = 35.7%, REML = 159.14, Scale est. = 1, *n =* 50.

Chosen model for abundance of nymphs explains a moderate portion of the variance in the data (adjusted *R*^2^ = −0.351) and explains 35.7% of the deviance, suggesting a moderate fit (Table 2, Fig. 4). A non-linear effect of elevation on number of nymphs is more pronounced on the south side (coefficient = 1.934, *p* < 0.001), but not on the opposite slope, and indicates highest abundance at middle elevation (S720, Fig. 4). Temperature has a substantial effect on nymph abundance on the south-facing slope only (coefficient = 2.167, *p* = 0.007), with increased abundance at higher temperatures (Fig. 4).

A separate analysis on adult males and females showed the following: in males (Supplementary File B, Supplementary File C), suggested model has moderate level of explanatory power (adjusted *R*^2^ = 0.526). On the south-facing slope, there was a significant linear relationship between temperature and abundance of males (coefficient = −0.023, *p* = 0.020). As temperature increases, the number of male ticks decreased. However, on the north-facing slope, the effect of temperature on number of males was better captured by a non-linear relationship (coefficient = 1.919, *p* = 0.007), and most adult males were predicted at temperatures between 16 and 17 °C (Supplementary File C). With further increases in temperature, the number of male ticks decreased. Elevation affected the number of males on the south-facing slope in a non-linear way (coefficient = 3.202, *p* = 0.005), with most ticks predicted at elevation 720 m (S720; Supplementary File C).

In females (Supplementary File B, Supplementary File C), the overall fit of the best model was modest (25.7% of deviance explained), explaining a small portion of the variability in the data (adjusted *R*^2^ = 0.138). Elevation had a marginal effect on the north-facing slope (coefficient = 1.769, *p* = 0.072), while it was significant on the south-facing slope (coefficient = 1.886, *p* = 0.034), again with most ticks predicted at elevations around sampling sites S720 in S820 (Supplementary File C).

## Discussion

In recent decades, a striking pattern has emerged in which many tick species are increasingly shifting their distribution areas northwards and upwards due to extensive climate changes [33]. The results of previous research on the vertical distribution of *I. ricinus* ticks in Central Europe indicate that climate change not only facilitates the survival of this species at higher elevations, but also enables them to complete their life cycle due to the resulting higher average temperatures [8, 13]. The elevational distribution limit of the tick *I. ricinus* in Central Europe, which used to be around 700–800 m above sea level [8, 29], has shifted significantly in the past two decades [8, 10].

We hypothesise that tick abundance on Mount Slavnik is higher at lower elevations, which correspond to a warmer climate. Additionally, we expect that the number of ticks increases in relation to the abundance of hosts. Although the population density of potential host species, particularly roe deer, may also have an influence on the distribution of ticks on Mount Slavnik, we did not include wildlife in our study.

Research conducted on nine hills in Scotland, all located in open moorland, found a positive correlation between tick abundance and deer population and a remarkably strong negative correlation between ticks and elevation [19]. In our study, we were able to demonstrate a significant influence of elevation, temperature, and slope orientation on the abundance of *I. ricinus* ticks on Mount Slavnik in the hinterland of the north-eastern Adriatic coast.

Regarding the differences in tick abundance (in both adults and nymphs) between the northern and southern slopes (N > S), our results suggest that *I. ricinus*, a species known to prefer closed forest habitats [18], more easily expands to higher elevations on the northern slope of the Mount Slavnik. This pattern is consistent with the observation that the forest habitat on the northern slope extends to higher elevations (to *ca.* 960 m) compared to the southern side of the mountain where a contiguous forest does not reach this elevation (only to *ca.* 850 m). On the southern slope, *I. ricinus*, however, was also found regularly at elevation of 920 m. It is noteworthy that site S920 on the southern slope is separated from the contiguous forest habitat that predominates at lower elevations on this side of the mountain. Nevertheless, a sole nymph collected at the highest sampling site (S1020) proved that in this area, *I. ricinus* is successfully expanding to the top of the mountain.

The overall greater number of ticks on the northern slope may also be attributed to the thicker layer of leaf litter that was present at all sampling sites, whereas on the southern slope, except for collection site S920, leaf litter was consistently sparse (as indicated in Table 1). Despite the occasional strong winds on the northern slope, the thicker leaf litter can be explained by the lower gradient, which reduces the effects of water runoff after heavy rainfall. On steeper slopes, rain tends to wash away organic material more quickly and prevent the accumulation of leaf litter. In contrast, foliage on the gentler, northern slope can settle and decompose more slowly, retaining moisture and creating a stable microhabitat for ticks. Winds may scatter some foliage, but may not be enough to compensate for the lower runoff and higher organic storage compared to the steeper south-facing slope, where faster water runoff and stronger sunlight accelerate decomposition.

The highest density (No. of individuals per 100 m^2^) of adult ticks (see also Table 1) was found at medium elevation on the northern slope (sampling site N720; average/median *=* 13.5/12.1 ticks) with the lowest average temperature (AVG ± SE: 16.7 °C ± 0.2 °C) and the highest average relative humidity (84.9% ± 0.6%). All other sampling sites had lower abundances of adult ticks, with the exception of two cases (S720 and N520). In contrast to site N720, however, the average temperatures recorded at sites S720 (18.1 °C ± 0.2 °C) and N520 (17.1 °C ± 0.2 °C) were higher (significant only for S720, *p* < 0.05: pairwise comparison, Bonferroni correction), while average relative humidity values were lower (S720: 75.1% ± 0.6%; N520: 81.9% ± 0.7%; significant only for S720, *p* < 0.05: pairwise comparison, Bonferroni correction). As indicated by the best negative binomial GAM model for the adults, middle elevations (700–800 m) and middle temperatures (approx. 17 °C; especially at the north-facing slope) in spring 2023 in the study area has a positive influence on adult tick abundance. This also applied for males alone; however, on the south-facing slope, their abundance decrease with increasing temperatures. For females, middle elevations are also preferred. Relative humidity, however, did not prove as an important factor in GAM models, but it should be noted that the relative humidity was high on both sides at middle elevations (Fig. 2). Compared to N720, lower abundances of adult ticks were found at the remaining sites. In addition, no adult ticks were collected at both the lowest average temperature and the lowest relative humidity at the highest site S1020. It appears that a combination of low spring temperature and humidity (average: *T* = 17.1 °C, RH = 71%) limits the higher abundance of ticks at this site. It should also be noted that at S1020, there was only a small and isolated stand of *Pinus nigra* and not of a mixed forest, which is known to be a favourable habitat type for *I. ricinus* [1], and that its small size at a wind-exposed position near the top of the mountain could negatively affect the abundance as well as the ontogenetic development of ticks. Field experience and data on the distribution of ticks in Czechia also indicate a remarkable change in the elevation at which these ticks occur [28]. In addition, the observed occurrence of tick populations at higher altitudes indicates that the geographical distribution area of *I. ricinus* in Central Europe is changing [9, 11, 13]. However, in the hinterland of the warm sub-Mediterranean region, *I. ricinus* was found at higher elevations than typically documented for its distribution area in continental Central Europe, and these ticks are often observed at higher elevations on the southern side than on the northern side [8, 28].

The highest adult tick abundance at N720 (and partly also at S720 and N520) is consistent with the habitat characteristics in these areas. The forest is densest at these sites (Fig. 1), providing a stable microclimate with higher humidity and reduced exposure to strong winds, which are crucial for tick survival. These favourable conditions likely facilitate the completion of the tick life cycle, despite earlier assumptions that elevations above 700 m were unsuitable for *I. ricinus*. While previous studies, particularly those on the ecological parameters of snowdrop populations in Central Europe, suggested that ticks would struggle to develop at such altitudes, more recent research confirms that *I. ricinus* regularly occurs at elevations exceeding 1 000 m [8, 9]. As an exophilic species, *I. ricinus* is sensitive to climatic conditions and requires a relative humidity of at least 80% to survive during the time it is not living on the host [30]. In our study area, this corresponds (in average values) only to elevations up to 820 m on the northern slope, while it is much lower at all other sites. While on the southern slope of Slavnik, the average relative humidity was always below 80% during the study period (*i.e.* about 70–75%), but increased with increasing elevation up to 820 m, the decrease in relative humidity was considerable at the two highest sites on this slope. The sampling site at 920 m (S920) was in an isolated patch of forest, and the highest sampling site (S1020) was in a small, isolated, and sparse *Pinus nigra* stand, both open to a neighbouring meadow exposed to the strong local bora wind (strongest at the top of the windward side of the slope) [40]. The very low number of ticks collected at the highest site (S1020) was expected. In contrast, the southern site at 920 m had the highest mean number of nymphs collected, likely due to the presence of a thick leaf litter layer, which provides ideal conditions for oviposition. Additionally, the absence of ground vegetation suitable for adult foraging [20] and the litter’s ability to retain moisture throughout the day [30] create a stable microhabitat with high humidity [25], further supporting the survival of the nymphs. The chosen GAM model for nymphs, however, indicated significant interactions of elevation and temperature with the southern side of the slope. Although the model’s fit to the data is not as good as for the adults, and much of the variability remains unexplained, it is consistent with the best model for the adults, as it also indicates highest abundance at middle elevations, especially on the southern slope (S720: 700–800 m). It should be noted, however, that the model suggests the abundance of nymphs is in positive correlation with the temperature, as the increased temperatures probably positively affect their development when humidity in the microenvironment is high enough, and there is no saturation deficit [21]. As the vast majority of nymphs were collected from the leaf litter, the microclimatic conditions regarding the appropriate combination of temperature and relative humidity there should be suitable for their survival. A study conducted in the Krkonoše Mountains in Czechia, at elevation range 600–1270 m, recorded the highest *I. ricinus* nymph abundance in mixed forests at lowest elevations between 600 and 800 m, with a mean density of 31 nymphs per hour of flagging and a maximum of 125 nymphs per hour, but also the presence of nymphs at altitudes up to around 1 200 m [28]. While the study shares several similarities with ours, we highlight altitude ranges specific to our study area. Given that the Krkonoše Mountains are located approximately 700 km north of Slavnik mountain, it is expected that peak tick abundance would occur at lower elevations there due to regional climatic differences. Taking into an account the same 100 m belts of elevation, the maximum in Krkonoše mountains for nymphs is lower (600–700 m) than in our study area in Slovenia (700–800 m). While nymph abundance decreased with elevation in Czechia, this was not the case in our study, where middle elevations (approx. between 700–800 m) had the highest abundances of ticks (nymphs and adults). Importantly, no data for elevations below 600 m are provided for the study from the Krkonoše mountains.

This study provides valuable data on *I. ricinus* in Slovenia’s Coastal-Karst region, a transition zone where Mediterranean, Alpine, and Central European climates meet, and improves our understanding of the ecological adaptability of this species in this complex microclimate, which has not been extensively studied so far. Our results are also consistent with ongoing European research [2] suggesting that *I. ricinus* is expanding its range to higher elevations, likely due to climate warming. In particular, the observed abundance at elevations above 700–800 m on Mount Slavnik mirrors trends observed in other regions, further supporting the ecological range expansion of *I. ricinus* [27].

The presence of ticks along the popular hiking trails of Mount Slavnik, which is visited by more than 10 000 hikers annually (unpublished data from the hut owner), can have a significant impact on public health. If the ticks are infected with pathogens such as *Borrelia burgdorferi* (Lyme disease) or TBE (tick-borne encephalitis virus), there is an increased risk of infections that could burden the health system and affect tourism. Preventive measures, such as educating hikers, putting up signs, and promoting the use of repellents as well as proper trail maintenance are crucial to reducing the risk. The planned analysis of tick-borne pathogens will be a crucial step in assessing the actual threat and developing further protective measures. Nevertheless, we believe that these results will contribute to a more comprehensive understanding of the bionomics of this tick species in Europe, both biologically and epidemiologically.

## Conclusion

This study highlights the significant influence of meteorological factors, elevation and slope orientation on the distribution and abundance of *I. ricinus* ticks on Mount Slavnik in Slovenia. The results show that tick abundance is highest at elevations between 700 and 800 m (but lower on the south-facing than the north-facing slope) where favourable microclimatic conditions support their survival. A northward distribution pattern is likely due to denser forests and thicker leaf litter, providing a stable microhabitat. Consistent with other European research, these findings suggest that *I. ricinus* is adapting to higher altitudes, which is likely influenced by climate change. This emphasizes the need for further research on bionomics and potential health implications across Europe.

## Figures and Tables

**Supplementary File C:**
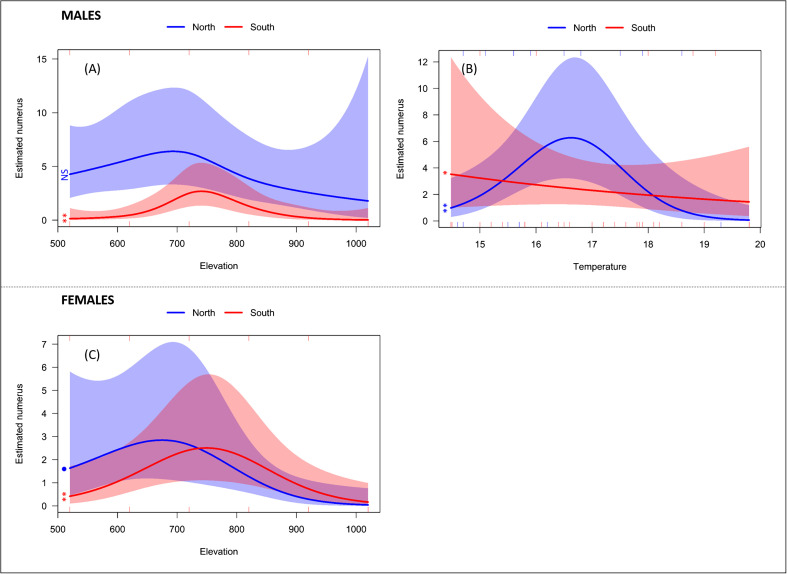
Smooth terms of a negative binomial generalised additive model for abundance of *Ixodes ricinus* males and females.

## Supplementary Files

*Supplementary File A*: Data on average temperature (T) and relative humidity (RH) at each sampling site during the study period. Average values ± standard error (standard deviation) are given. *Supplementary File B*: Chosen general additive model (GAM with negative binomial function) for males and females showing the interaction effect between side of the slope, elevation, and temperature in terms of number of collected adult *Ixodes ricinus*.

The supplementary material for this article can be found at https://www.parasite-journal.org/10.1051/parasite/2025044/olm.
