# Supplementary material for: Spatial distribution of Ixodes ricinus in forest habitats: a comparative study of the northern and southern slopes of Mount Slavnik, Slovenia
Source: Parasite. 2025 Jul 25;32:46. doi: 10.1051/parasite/2025044 (PMC12291548; doi:10.1051/parasite/2025044)
Supplement: Supplementary file 1 — Supplementary File A: Data on average temperature (T) and relative humidity (RH) at each sampling site during the study period. Average values ± standard error (standard deviation) are given. [file parasite-32-46-s1.pdf]

Supplementary File A.

Data on average temperature (T) and relative humidity (RH) at each sampling site during the study period. Average values ± standard error (standard deviation) are given.

Sampling site	T (ºC)	RH (%)
S520	19.88 ± 0.16 (4.62)	73.17 ± 0.69 (19.39)
S620	19.02 ± 0.15 (4.32)	74.15 ± 0.64 (17.95)
S720	18.13 ± 0.15 (4.14)	75.13 ± 0.63 (17.57)
S820	17.26 ± 0.15 (4.11)	76.08 ± 0.65 (18.31)
S920	17.27 ± 0.17 (4.67)	70.17 ± 0.78 (21.71)
S1020	17.08 ± 0.16 (4.52)	70.53 ± 0.72 (20.22)
N920	16.95 ± 0.15 (4.25)	75.44 ± 0.64 (17.80)
N820	16.83 ± 0.15 (4.11)	80.19 ± 0.61 (16.96)
N720	16.70 ± 0.15 (4.10)	84.93 ± 0.64 (17.81)
N620	18.90 ± 0.23 (6.48)	85.19 ± 0.66 (18.53)
N520	17.10 ± 0.17 (4.82)	81.87 ± 0.71 (19.76)
