# Supplementary material for: Spatial distribution of Ixodes ricinus in forest habitats: a comparative study of the northern and southern slopes of Mount Slavnik, Slovenia
Source: Parasite. 2025 Jul 25;32:46. doi: 10.1051/parasite/2025044 (PMC12291548; doi:10.1051/parasite/2025044)
Supplement: Supplementary file 2 — Supplementary File B: Chosen general additive model (GAM with negative binomial function) for males and females showing the interaction effect between side of the slope, elevation, and temperature in terms of number of collected adult Ixodes ricinus. [file parasite-32-46-s2.pdf]

Supplementary File B.

Chosen general additive model (GAM with negative binomial function) for males and females showing the interaction effect between side of the slope, elevation and temperature in terms of number of collected adult *Ixodes ricinus*. Significant p values ($p < 0.05$) in bold. *** $p < 0.001$, ** $p < 0.01$, * $p < 0.05$, ● $p < 0.1$, NS non-significant.

Males	*Parametric coefficients*	*Estimate*	*Std. error*	*z value*	*p*	
	Intercept	0.000	0.000	NaN	NaN	
	Temperature × Side of slope - North	0.023	0.017	1.336	0.182	NS
	Temperature × Side of slope - South	-0.059	0.025	-2.332	**0.020**	*
	Approx. significance of smooth terms	*edf*	*Ref. df*	χ^2	*p*	
	Elevation × Side of slope - North	2.102	2.543	2.082	0.378	NS
	Elevation × Side of slope - South	3.202	3.878	15.484	**0.005**	**
	Temperature × Side of slope - North	1.919	1.993	10.234	**0.007**	**
	Temperature × Side of slope - South	1.000	1.000	0.253	0.615	NS
Females	*Parametric coefficients*	*Estimate*	*Std. error*	*z value*	*p*	
	Intercept	-0.062	0.266	-0.233	0.816	NS
	Approx. significance of smooth terms	*edf*	*Ref. df*	χ^2	*p*	
	Elevation × Side of slope - North	1.769	1.947	4.542	0.072	•
	Elevation × Side of slope - South	1.886	1.987	6.588	**0.034**	*

Males: R^2 (adj) = 0.526, Deviance explained = 65.3%, -REML = 71.963, Scale est. = 1.0151, n = 50; Females: R^2 (adj) = 0.138, Deviance explained = 25.7%, -REML = 76.097, Scale est. = 1, n = 50
